# Empagliflozin Perception Mapping Survey

**DOI:** 10.7759/cureus.99024

**Published:** 2025-12-12

**Authors:** Rajiv Kovil, Vijay Panikar, Jothydev Kesavadev, Snehal Tanna, Kamlesh Nayak, Dharmen Punatar, Lotika Purohit, Arun Kumar Kedia, Ami Sanghvi, Aravinda Jagadeesha, Sameer Chandratre, Sanhita Walawalkar, Mahendra Patel, Anil Kumar Virmani, Girish Mathur, Ketan K Mehta, Sameer Muchhala, Vishal Gala, Akanksha Sonkar

**Affiliations:** 1 Diabetes and Endocrinology, Zandra Healthcare, Mumbai, IND; 2 Diabetes and Endocrinology, Lilavati Hospital, Mumbai, IND; 3 Diabetes and Endocrinology, Jothydev’s Diabetes Research Centre, Thiruvananthapuram, IND; 4 Diabetes and Endocrinology, Jupiter Hospital, Thane, IND; 5 Diabetes and Endocrinology, Trident Hospital, Mumbai, IND; 6 Diabetes and Endocrinology, Diabcare Centre, Mumbai, IND; 7 Diabetes and Endocrinology, SugarDoctor Medicare LLP, Mumbai, IND; 8 Internal Medicine, Lifeworth Hospital, Raipur, IND; 9 Diabetes and Endocrinology, Sanghvi Eye and Diabetes Care Centre, Mumbai, IND; 10 Internal Medicine, Dr Aravind's Diabetes Centre, Bangalore, IND; 11 Pulmonology, Aarogyam Clinic, Nashik, IND; 12 Diabetes and Endocrinology, Dr Panikar’s Diabetes and Thyroid Care Centre, Mumbai, IND; 13 Diabetes and Endocrinology, Patel Diabetes Care Centre, Thane, IND; 14 Internal Medicine, Viru's Diabetes and Cardiac Care Centre, Jamshedpur, IND; 15 Diabetes and Endocrinology, Alka Diagnostic Centre, Kota, IND; 16 Medicine, Suchak Hospital and Research Centre, Mumbai, IND; 17 Medical Affairs, Zydus Lifesciences Limited, Mumbai, IND; 18 Medical Affairs, Zydus Healthcare Limited, Mumbai, IND

**Keywords:** chronic kidney disease, empagliflozin, heart failure, physician perceptions, sglt2 inhibitors, type 2 diabetes mellitus

## Abstract

Background: Heart failure (HF) is a frequent yet often underdiagnosed comorbidity among individuals with type 2 diabetes mellitus (T2DM). Sodium-glucose cotransporter-2 (SGLT2) inhibitors, particularly empagliflozin, are known to provide significant cardiovascular and renal benefits and are endorsed by international guidelines. However, real-world gaps in adoption persist, influenced by diagnostic challenges, cost considerations, and clinician familiarity. This survey assessed physician perceptions and prescribing practices regarding empagliflozin across diverse T2DM patient profiles in India.

Methods: A cross-sectional, questionnaire-based survey was conducted using a voluntary convenience sampling approach among physicians from various specialities managing T2DM. As the study involved anonymized physician responses without patient-level data, ethical approval was not applicable. The survey captured information on demographic characteristics, heart failure risk assessment, SGLT2 inhibitor prescribing patterns, perceived cardio-renal benefits, and perceived barriers. Data were analyzed descriptively and summarized as frequencies and proportions.

Results: Most respondents acknowledged that HF remains underdiagnosed in T2DM and recognized the role of empagliflozin in patients with cardiovascular and renal comorbidities. Physicians reported frequent use of empagliflozin in T2DM with multiple cardiovascular risk factors, established atherosclerotic cardiovascular disease (ASCVD), and chronic kidney disease (CKD). Recent evidence supporting its benefit in both, heart failure with reduced ejection fraction (HFrEF) and heart failure with preserved ejection fraction (HFpEF) was reflected in prescribing trends. Cost, guideline awareness, and therapeutic inertia were cited as continuing barriers to optimal utilization.

Conclusions: Indian physicians demonstrate strong awareness and largely guideline-concordant prescribing attitudes toward empagliflozin in T2DM with cardiovascular or renal complications. Nevertheless, under-recognition of asymptomatic HF and variability in CKD management highlight the need for targeted education and policy initiatives to further strengthen evidence-based practice.

## Introduction

In Individuals with type 2 diabetes mellitus (T2DM), heart failure (HF) is a common but often underdiagnosed comorbidity [1]. According to recent estimates, more than 537 million adults worldwide suffer from diabetes, and the disease prevalence is still rising [2]. India is one of the nations with the highest prevalence of diabetes, accounting for about 77 million of these cases [3]. An estimated 9% to 22% of patients with type 2 diabetes have heart failure, and even in the absence of a history of myocardial infarction or structural heart disease, the risk of developing heart failure is much higher in this population [4]. Crucially, because of overlapping symptoms and diagnostic complexity, a significant percentage of people with type 2 diabetes may have asymptomatic or undetected heart failure, especially heart failure with preserved ejection fraction (HFpEF) [5-7]. Furthermore, up to 40% and 25% of individuals with type 2 diabetes, respectively, have comorbid conditions like chronic kidney disease (CKD) and atherosclerotic cardiovascular disease (ASCVD), which makes management of the disease difficult and hence increases the risk of cardiovascular disease [8].

Sodium-glucose cotransporter-2 (SGLT2) inhibitors have emerged as an important component of the therapeutic armamentarium for T2DM, especially in patients with cardiovascular or renal comorbidities [9]. These agents work by blocking SGLT2 in the proximal renal tubule, which in turn reduces the kidney’s reabsorption of glucose and sodium. This mechanism leads to increased urinary glucose excretion, a modest degree of natriuresis, and a lowering of intraglomerular pressure [10,11]. One of these agents is empagliflozin, which is now recommended for patients with T2DM who also have coexisting cardiovascular or renal disease, according to guidelines from the American Diabetes Association (ADA) and Kidney Disease Improving Global Outcomes (KDIGO) [12,13]. In addition, there remain gaps in practical application despite this strong clinical evidence. Widespread adoption may be hampered by factors like cost, genitourinary infection risk, unfamiliarity with updated guidelines, and therapeutic inertia, according to reports from clinician surveys [14]. The Empagliflozin perception mapping survey aimed to evaluate physician perspectives on the use of empagliflozin in diverse clinical scenarios involving T2DM, with a focus on cardiovascular and renal risk profiles, heart failure management, and practical prescribing challenges.

Therefore, the present study aimed to evaluate physician perceptions and prescribing practices related to empagliflozin across diverse clinical scenarios in T2DM, with particular emphasis on cardiovascular risk, CKD, and heart failure management.

## Materials and methods

Study design and participants

A cross-sectional survey was conducted to evaluate physician perspectives and prescribing practices regarding the use of empagliflozin in T2DM across diverse clinical scenarios. A total of 129 physicians were recruited using purposive sampling during advisory board meetings held between February and April 2025. Eligible participants included practising diabetologists, consulting physicians, cardiologists, and endocrinologists who manage patients with T2DM. Participation was voluntary, and physicians were invited to complete a structured questionnaire during the meetings or immediately thereafter via a secure survey link.

Survey instrument

The questionnaire consisted of 13 items developed specifically for this study. The survey underwent an internal two-step validation process (Appendix A).

Content Validation

Content validation was conducted by the study moderator and two independent clinical experts, who reviewed all items for clarity, relevance, and alignment with real-world practice.

Pilot Testing

The draft survey was piloted with five physicians to ensure clarity and usability. No major revisions were required. The questionnaire was organized into five clinically relevant domains: Participant demographics, including medical speciality and years of clinical experience; Physician assessment of heart failure prevalence and risk in the T2DM population; Selection preferences for SGLT2 inhibitors across different patient profiles; Utilization of empagliflozin in the management of heart failure; Identification of adverse effects and barriers to prescribing encountered in practice.

The survey included single-choice, multiple-choice, and Likert-scale questions, with response options ranging from strongly agree to strongly disagree, allowing for the capture of nuanced clinical perspectives and varying degrees of agreement.

Survey Administration and Data Collection 

Data were collected from February to April 2025 using a secure, web-based survey platform. Participants submitted anonymized responses electronically. No identifying information was collected. All completed surveys were exported to a password-protected database for analysis.

Statistical analysis 

Survey responses were collected and subjected to descriptive statistical analysis. Categorical variables were reported as frequencies and percentages to characterize response patterns. The analysist examined physician perceptions of empagliflozin's cardiovascular protective effects, renal benefits, and heart failure management efficacy across different patient populations encountered in clinical practice.

Ethical considerations

This study involved a voluntary, anonymized, opinion-based survey of licensed physicians and did not include any patient data, personal identifiers, or sensitive personal information. The questionnaire captured only professional practice perspectives related to prescribing patterns and clinical decision-making. According to institutional policy and prevailing national norms for minimal-risk research, surveys of healthcare professionals that do not collect identifiable information and do not involve patients are classified as exempt from formal ethics committee review. Therefore, this study met the criteria for exemption, and separate ethics committee approval was not required. Completion of the survey constituted implied consent for participation.

## Results

A total of 129 physicians participated in the survey. The majority of respondents were diabetologists (78, 60.5%), followed by consulting physicians (45, 34.9%). Cardiologists and endocrinologists each comprised 2.3% of the sample (3 participants each). The physician cohort was highly experienced, with 77 (59.7%) having more than 20 years of clinical practice. Those with 11-20 years of experience represented 38 (29.5%), while physicians with 5-10 years and less than 5 years of experience comprised 11 (8.5% ) and 3 (2.3%), respectively.

**Figure 1:**
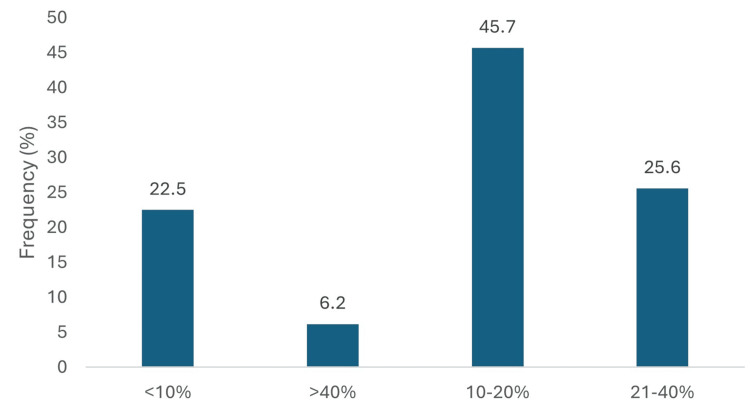
Percentage of T2DM with undiagnosed heart failure. Data has been presented as %.

When asked about the prevalence of undiagnosed heart failure among the T2DM, 59 (45.7%) estimated the rate to be 10-20%, while 33 (25.6%) believed it ranged from 21-40%. Only 8 (6.2%) suspected a prevalence above 40%, and 29 (22.5%) physicians estimated it to be below 10% (Figure 1).

**Figure 2:**
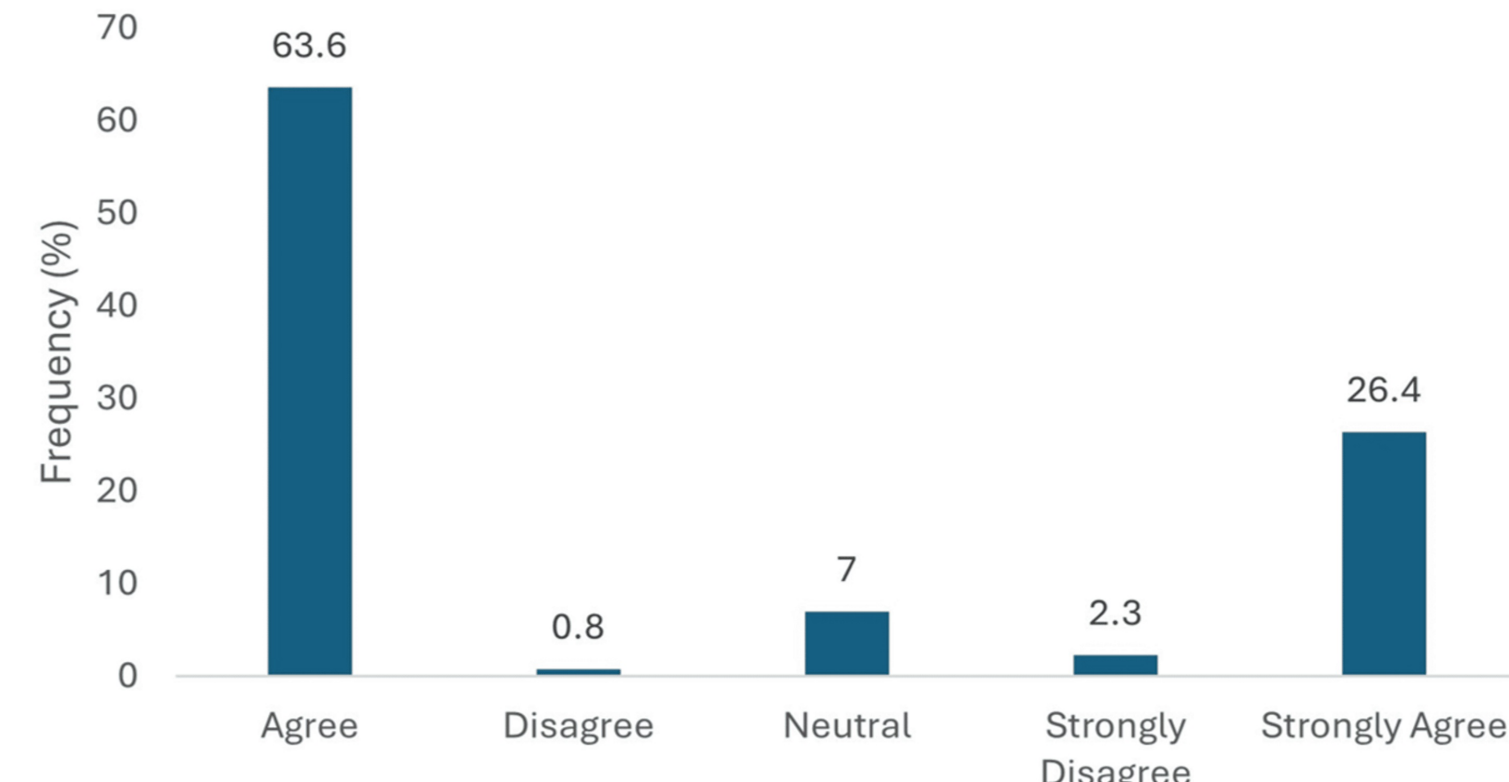
Heart failure underdiagnosed in T2DM. Data has been presented as %.

A substantial majority of physicians (116, 90.0%) believed that heart failure is underdiagnosed in T2DM patients, with 82 (63.6%) agreeing and 34 (26.4%) strongly agreeing with this statement. Only 1 (0.8%) disagreed, while 9 (7.0%) remained neutral and 3 (2.3%) strongly disagreed (Figure 2).

**Figure 3:**
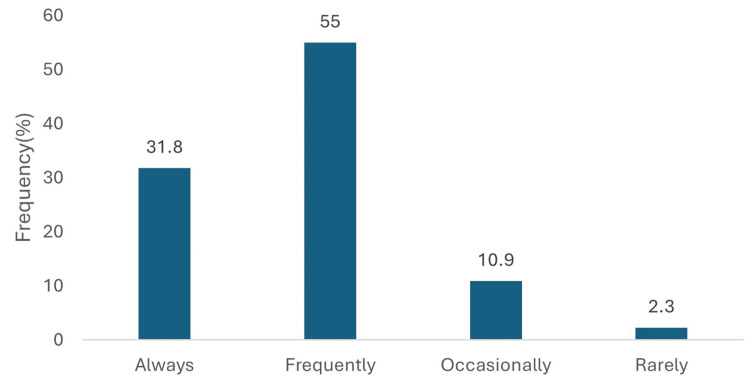
Empagliflozin for T2DM with multiple cardiovascular risk factors. Data has been presented as %.

Among patients with T2DM who had multiple cardiovascular risk factors but no established atherosclerotic cardiovascular disease (ASCVD), 112 (86.8%) of physicians reported prescribing empagliflozin either frequently or always. Of these, 71 (55.0%) indicated that they frequently prescribed the medication, while 41 (31.8%) reported always prescribing it. In contrast, 14 (10.9%) prescribed it only occasionally, and 3 (2.3%) rarely used empagliflozin in this patient group (Figure 3).

**Figure 4:**
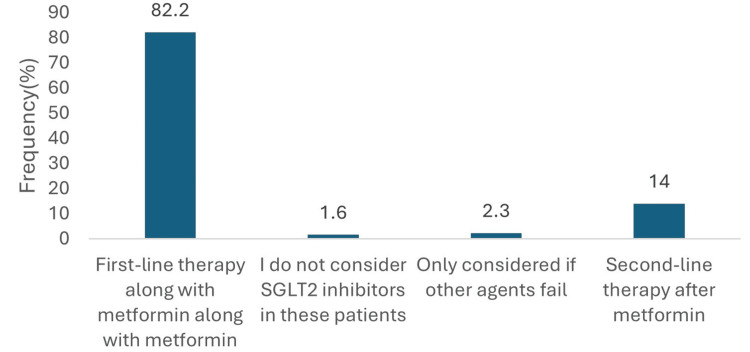
Empagliflozin treatment preference ranking T2DM with ASCVD. Data has been presented as %.

In patients with established ASCVD, the majority of physicians (106, 82.2%) reported prescribing empagliflozin as first-line therapy in combination with metformin. A smaller proportion (18, 14.0%) considered it as a second-line option following metformin. Very few physicians prescribed it only after failure of alternative treatments (3, 2.3%), and 2 (1.6%) did not consider SGLT2 inhibitors for this population (Figure 4).

**Figure 5:**
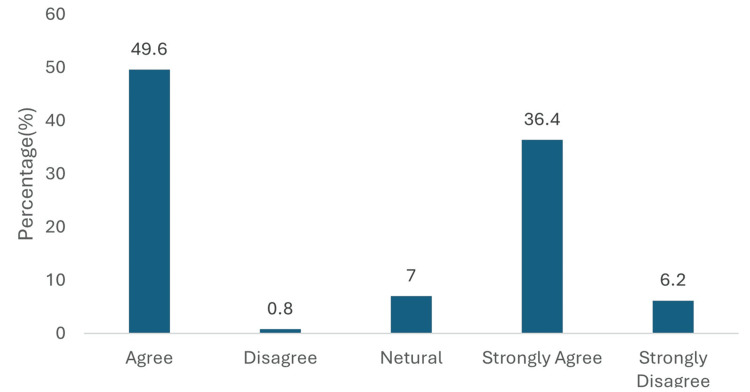
Perceived role of empagliflozin in delaying CKD progression. Data has been presented as %.

Regarding empagliflozin's role in T2DM patients with chronic kidney disease (CKD) and proteinuria, physicians demonstrated strong confidence in its renal protective effects. For delaying CKD progression, 111 (86.0%) expressed positive views, with 64 (49.6%) agreeing and 47 (36.4%) strongly agreeing. Only 9 (7.0%) remained neutral, while disagreement was minimal (1, 0.8% disagreed; 8, 6.2% strongly disagreed) (Figure 5).

**Figure 6:**
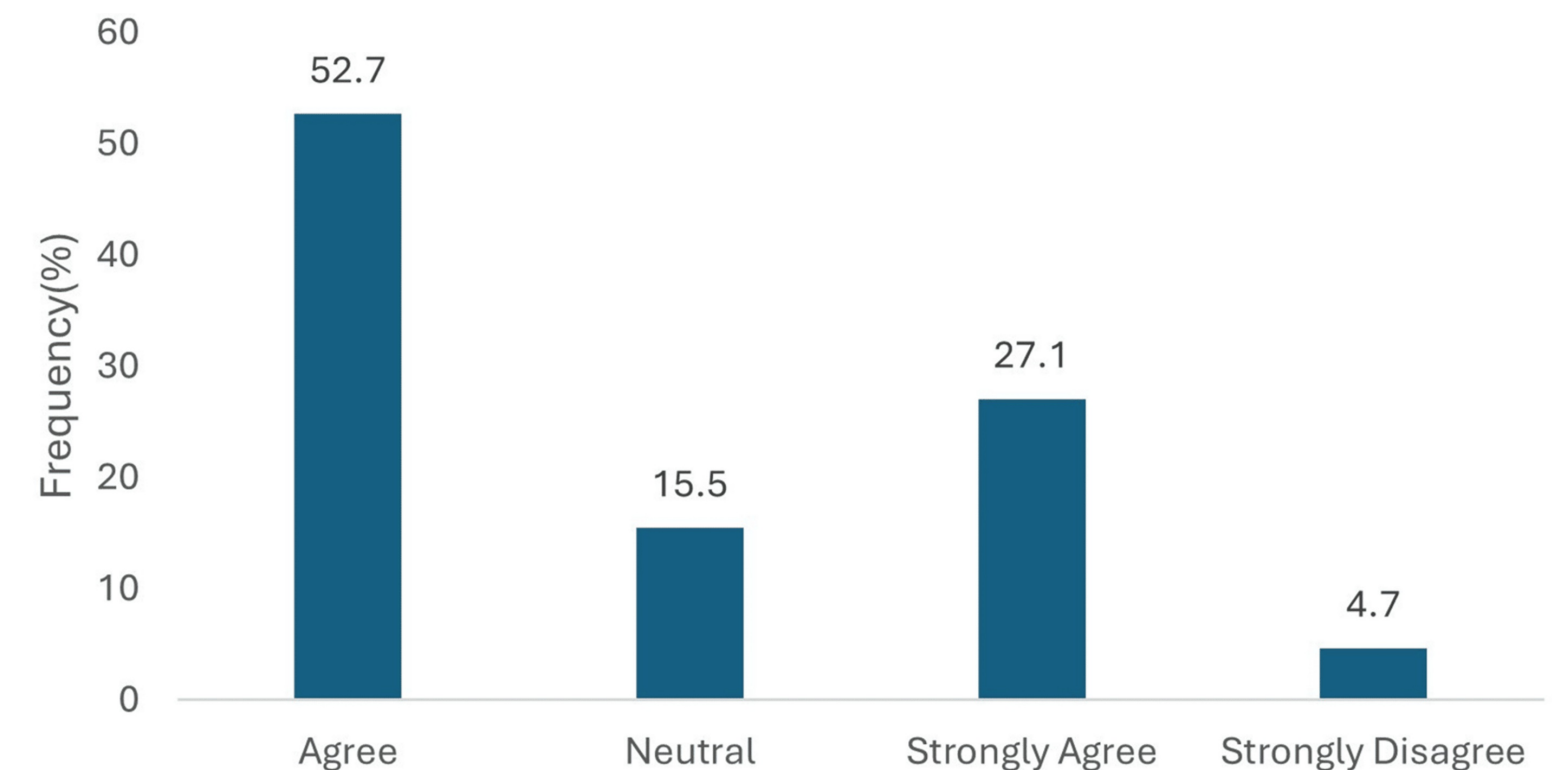
Perceived role of empagliflozin in reducing proteinuria in T2DM patients. Data has been presented as %.

Similarly, for proteinuria reduction, 103 (79.8%) physicians responded positively, with 68 (52.7%) agreeing and 35 (27.1%) strongly agreeing, 20 (15.5%) remained neutral, and only 6 (4.7%) strongly disagreed. Physicians overwhelmingly rejected the notion that empagliflozin provides no significant renal benefit. A total of 109 (84.5%) disagreed with this statement, with 55 (42.6%) disagreeing and 54 (41.9%) strongly disagreeing. Only 6 (4.7%) expressed agreement (5, 3.9% agreed; 1, 0.8% strongly agreed) (Figure 6).

**Figure 7:**
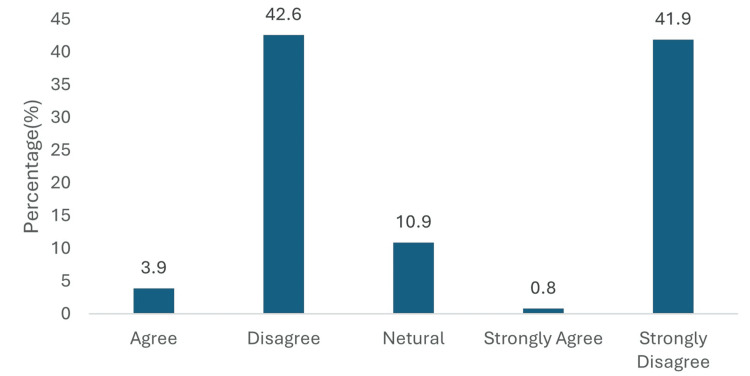
Perceived role of empagliflozin in reducing proteinuria in T2DM patients with CKD progression. Data has been presented as %.

Physicians overwhelmingly rejected the notion that empagliflozin provides no significant renal benefit. A total of 84.5% disagreed with this statement, with 55 physicians (42.6%) disagreeing and 54 physicians (41.9%) strongly disagreeing. Only 6 physicians (4.7%) expressed agreement (5, 3.9% agreed; 1, 0.8% strongly agreed) (Figure 7).

**Figure 8:**
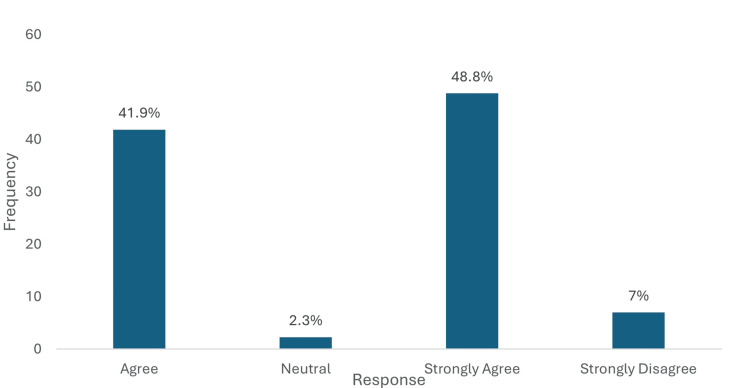
Perceptions regarding empagliflozin's cardiovascular benefits in Type 2 diabetes patients with CKD and proteinuria. Data has been presented as %.

A substantial majority of physicians (117, 90.7%) expressed positive perceptions regarding empagliflozin's cardiovascular benefits in T2DM with CKD and proteinuria. Specifically, 54 (41.9%) agreed, and 63 (48.8%) strongly agreed with the cardiovascular benefits. Only 9 (7.0%) strongly disagreed, while 3 (2.3%) remained neutral (Figure 8).

**Figure 9:**
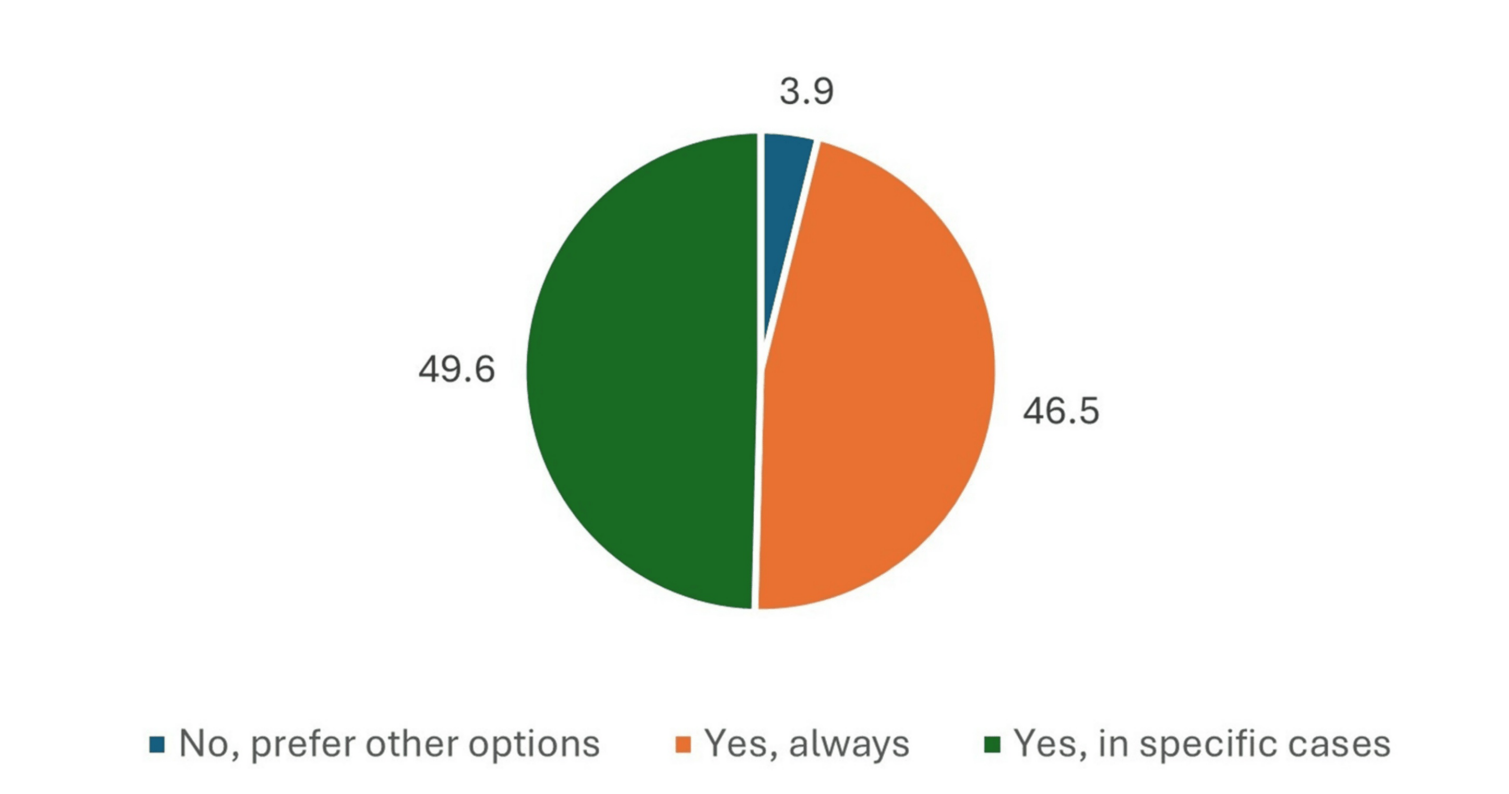
Empagliflozin prescribing decision in non-proteinuric CKD patients with T2DM. Data has been presented as %.

When considering T2DM patients with CKD but without proteinuria, 124(96.1%) of physicians would prescribe empagliflozin. The majority (64, 49.6%) indicated they would prescribe it in specific cases, while 60 (46.5%) reported they would always prescribe it. Only 5 (3.9%) preferred other treatment options (Figure 9).

**Figure 10:**
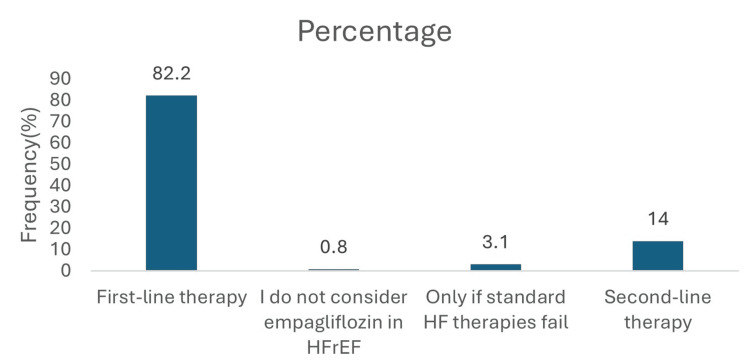
Empagliflozin priority in T2DM with HFrEF; EF>40%. Data has been presented as %.

In T2DM patients with HFrEF: EF >40%, 106 (82.2%) prioritized empagliflozin as first-line therapy. An additional 18 (14.0%) considered it as second-line therapy. Only a small minority indicated its use solely after failure of standard heart failure therapies (4, 3.1%) or did not consider its use at all (1, 0.8%) (Figure 10).

**Figure 11:**
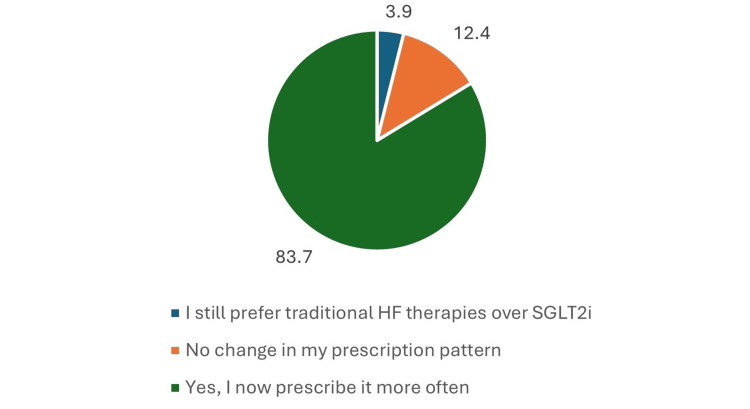
Change in prescribing behaviour (empagliflozin in HFpEF+T2DM). Data has been presented as %.

For patients with heart failure with HFpEF: EF >50%, recent clinical evidence significantly influenced prescribing patterns. Among all participants, 108 physicians (83.7%) reported that recent clinical evidence led them to prescribe empagliflozin more frequently. In contrast, 16 (12.4%) noted no change in their prescribing habits, and only 5 (3.9%) continued to prefer traditional heart failure therapies over SGLT2 inhibitors (Figure 11).

## Discussion

This cross-sectional survey provides a real-world evaluation of physician perspectives on the use of empagliflozin across a range of clinical scenarios in patients with T2DM. The present findings indicate a high level of awareness among Indian clinicians about the cardiovascular and renal benefits of empagliflozin, especially in high-risk subgroups such as those with atherosclerotic cardiovascular disease (ASCVD), HF, and CKD. Nonetheless, our results also highlight critical perceptual and diagnostic gaps, particularly in the recognition of undiagnosed HF and the adoption of empagliflozin in CKD without proteinuria. These findings point to areas where further clinical education and implementation support may be necessary. However, these encouraging trends coexist with areas where clinical practice still falls short of current evidence, particularly in the early recognition of heart failure and consistent adoption of SGLT2 inhibitors in CKD without proteinuria.

Heart failure is increasingly recognized as a major comorbidity in T2DM, with prevalence estimates ranging from 9% to 22% in observational studies, even in the absence of overt cardiovascular disease or myocardial infarction history [12]. In line with these observations, the majority of respondents, 116 (90%), in our survey agreed that HF is underdiagnosed in T2DM populations. However, only a small proportion (8, 6.2%) estimated that more than 40% of their own patients might have undiagnosed HF. This discrepancy indicates that although physicians acknowledge HF under-recognition at a population level, they may underestimate its true burden in their own practice, a pattern previously observed in real-world cohorts where HF prevalence exceeded clinician expectations. Such findings echo prior real-world data, such as that reported byStoyanova et al., where HF prevalence among T2DM patients in a large German cohort exceeded 15%, yet initiation rates for evidence-based HF therapies remained suboptimal [1].

Most physicians in our study demonstrated guideline-concordant prescribing patterns for empagliflozin. In patients with ASCVD, 106 (82.2%) reported initiating empagliflozin as first-line therapy alongside metformin, consistent with the 2025 ADA Standards of Care, which recommend SGLT2 inhibitors with proven cardiovascular benefit irrespective of glycemic control [12]. The strong evidence base from the EMPA-REG OUTCOME trial and subsequent meta-analyses appears to have influenced these prescribing preferences, contributing to the widespread first-line positioning of empagliflozin reported by participants [15].

 A majority (111, 86.0%) agreed that it delays CKD progression, and 103(79.8%) endorsed its role in proteinuria reduction. Notably, 124 (96.1%) indicated they would consider prescribing it in CKD patients without proteinuria, reflecting awareness of recent guideline updates, such as those from KDIGO 2022 and 2024 guideline updates, which recommend SGLT2 inhibitors based on eGFR thresholds rather than albuminuria status alone [16]. However, published registry data from Canada and other global settings suggest that actual prescribing rates remain lower, particularly in non-proteinuric CKD. Despite this alignment, several international registry studies have documented low real-world uptake of SGLT2 inhibitors among eligible patients, suggesting that familiarity with evidence does not necessarily translate into routine prescribing [17,18]. 

In the context of heart failure, most survey respondents reported a preference for empagliflozin as part of first-line therapy in patients with HFrEF, with 106 (82.2%) indicating its routine initiation in this population. This is consistent with ESC 2021 HF guidelines (reinforced by the 2023 focused update) and the ACC/AHA/HFSA 2022 HF guidelines, which position SGLT2 inhibitors as a component of foundational pharmacotherapy for HFrEF regardless of glycemic status [19]. Notably, 108 (83.7%) of surveyed physicians indicated that their prescribing of empagliflozin for HFpEF had increased following recent trial evidence. This reported change aligns with emerging data demonstrating significant reductions in HF hospitalizations and improvements in symptom burden among T2DM patients with preserved ejection fraction, even in the absence of substantial glucose-lowering effects. Nevertheless, observational analyses such as that by Abudureyimu et al. suggest that real-world uptake in HFpEF remains lower than expected, highlighting the importance of ongoing dissemination of trial findings and updated guideline recommendations [8].

While our survey focused on physician perceptions rather than actual prescription data, these findings can be better understood within broader implementation science frameworks. For example, Yi et al. applied the Consolidated Framework for Implementation Research (CFIR), identified multiple barriers to SGLT2 inhibitor uptake, including medication cost, incomplete guideline familiarity, concerns about adverse effects (such as genitourinary infections), and therapeutic inertia [14].

Our survey findings indicate that physicians are increasingly aligning their reported prescribing preferences with contemporary evidence supporting empagliflozin’s role in T2DM patients with cardiovascular and renal comorbidities. Nonetheless, the responses also suggest ongoing diagnostic caution in the detection of asymptomatic HF, some reluctance to routinely initiate therapy in normoalbuminuric CKD, and the persistence of systemic barriers such as cost and access. Addressing these gaps will likely require a combination of targeted educational initiatives, streamlined dissemination of updated guideline recommendations, and policy-level measures aimed at improving equitable availability of SGLT2 inhibitors across diverse clinical settings.

Being a cross-sectional, self-reported survey, the findings reflect physician perceptions at a single point in time and may be subject to recall and response bias. The modest sample size and unequal representation across specialities and geographic regions may limit the generalizability of the results to the wider clinician population. As the survey relied on stated practices rather than actual prescription data, discrepancies between perceived and real-world prescribing behavior cannot be excluded. 

## Conclusions

This nationwide survey provides insight into how physicians perceive the role of empagliflozin in managing Type 2 diabetes mellitus, particularly in individuals with cardiovascular or renal comorbidities. Respondents expressed confidence in its cardiorenal benefits, and many reported using it routinely in settings such as ASCVD, HFrEF, and proteinuric CKD. However, because the survey captured self-reported perceptions rather than actual prescribing data, these responses may not fully reflect real-world practice patterns. The findings also point to several areas where clinical practice may not yet be fully aligned with evidence, including the under-recognition of asymptomatic heart failure, inconsistent adoption in non-proteinuric CKD, and practical challenges such as cost, access, and awareness of evolving guidelines. Targeted educational initiatives, better diagnostic pathways, and supportive policy measures may help narrow these gaps and promote more consistent evidence-based use of SGLT2 inhibitors in routine diabetes care.

## Appendices

Appendix A

**Table 1:** Empagliflozin perception mapping survey questionnaire. ASCVD: atherosclerotic cardiovascular disease, GU: genitourinary, CKD: chronic kidney disease, SGLT2: sodium-glucose cotransporter 2, HF: heart failure, T2DM: type 2 diabetes mellitus, CV: cardiovascular.

Section	Question	Response options
Section 1: Demographics	1. Your specialty	Endocrinology; Cardiology; Nephrology; Internal Medicine; Diabetology; Other (specify)
	2. Years of clinical practice	<5 years; 5–10 years; 11–20 years; >20 years
Section 2: Prevalence and risk perception of heart failure in type 2 diabetes	3. Suspected percent of undiagnosed HF in T2DM patients	<10%; 10–20%; 21–40%; >40%
	4. HF is underdiagnosed in T2DM patients	Likert scale: Strongly Agree; Agree; Neutral; Disagree; Strongly Disagree
Section 3: SGLT2 inhibitor preference in different patient profiles	5. Factors influencing SGLT2i choice in overweight T2DM patients	(Multiple choice) Weight loss benefit; Glycemic control; Cardiovascular protection; Renal protection; Cost/affordability; Risk of GU infections
	6. Frequency of initiating empagliflozin in T2DM with CV risk factors (no ASCVD)	Always; Frequently; Occasionally; Rarely
	7. Perceived role of empagliflozin in T2DM with CKD + proteinuria	Likert scale: Delays CKD progression; Reduces proteinuria; Provides cardiovascular benefits; No significant renal benefit
	8. Prescribing empagliflozin in CKD without proteinuria	Yes, always; Yes, in specific cases; No, prefer other options
	9. Empagliflozin ranking in T2DM with ASCVD	First-line with metformin; Second-line after metformin; Only if other agents fail; Do not consider SGLT2 inhibitors
Section 4: Empagliflozin use in heart failure	10. Prioritization of empagliflozin in T2DM with HFrEF (EF <40%)	First-line; Second-line; Only if standard HF therapies fail; Do not consider empagliflozin
	11. Change in empagliflozin prescription in HFpEF (EF >50%) after new evidence	Yes, prescribe more often; No change; Prefer traditional HF therapy over SGLT2 inhibitors
Section 5: Managing side effects and barriers to use	12. Approach for recurrent genital/urinary infections on SGLT2 inhibitors	(Multiple choice) Counsel on hygiene; Reduce dose; Switch therapy; Continue therapy
	13. Barriers to empagliflozin adoption	(Multiple choice) Cost; Risk of genital infections; Lack of awareness/clinical inertia; Concerns about eGFR decline; No significant barriers
